# Histologic Changes of Implanted Gore Bio-A in an Experimental Animal Model

**DOI:** 10.1155/2014/167962

**Published:** 2014-05-21

**Authors:** Kwan Koo Yeo, Tae Hwan Park, Jin Hyuk Park, Choong Hyun Chang, June-kyu Kim, Sang Won Seo

**Affiliations:** ^1^Department of Plastic and Reconstructive Surgery, College of Medicine, Kangbuk Samsung Hospital, Sungkyunkwan University School of Medicine, 108 Pyung-Dong, Jongno-gu, Seoul 110-746, Republic of Korea; ^2^Miz Aesthetic Surgery Clinic, 513-4 Shinsa-dong, Gangnam-Gu, Seoul 135-887, Republic of Korea

## Abstract

Gore Bio-A has been reported to be an ideal synthetic bioabsorbable scaffold material for hernia repair. The purpose of this study was to determine the effectiveness of Gore Bio-A in soft tissue augmentation. Six New Zealand white rabbits were used in the study. Five subcutaneous pockets were created on the back of the rabbit, and 20 × 20 mm sized square shaped Gore Bio-A sheets, each 1.5 mm, 3 mm, 4.5 mm, 6 mm, and 7.5 mm in thickness, were implanted into each pocket (1 layer to 5 layers). To analyze the morphologic and histologic changes, the implants were harvested 1, 3, and 6 months after implantation. Following the gross analysis, absorption rate was accelerated with increased implant duration and decreased thickness. Histological analysis of the implants demonstrated progressive neovascularization, fibroblast infiltration, and neocollagenation over time. Six months after implantation, Gore Bio-A was almost absorbed and degenerated, not maintaining its volume. Based on this study, Gore Bio-A was revealed as a biocompatible material; however, it is not suitable for soft tissue augmentation because it is absorbed in the process of changing into soft tissue without maintaining its own volume. Therefore, this material is incomplete and needs more study to overcome this limitation.

## 1. Introduction

Plastic surgeons are performing various types of soft tissue augmentation surgery for restorative or aesthetic purposes on disfigured body parts, including the face. Several materials such as autogenous, homogenous, and alloplastic materials can replace soft tissue [1–8]. Autologous tissues are considered ideal, but they have some disadvantages that involve additional operations such as the need for surgery for harvesting the tissue, the limited harvesting site, and the unguaranteed outcome due to absorption or disfigurement after the surgery [1, 2]. As such, continuous efforts have been made to find safe and effective alternative materials that do not have any side effects, and a number of artificial materials have been developed and used so far.

Gore Bio-A (WL Gore & Assoc, Flagstaff, AZ, USA) is a porous substance like Gore-Tex and has been developed recently and its use in anal fistula and hernia treatment has been reported [9–12]. The authors anticipated that Gore Bio-A would have potential as a soft tissue implant substituting for the weaknesses of existing substances and initiated research.

This study aims to investigate the changes in the histological pattern of an overlapping Gore Bio-A graft, particularly in absorption, and the changes in the soft tissue and the volume change in the grafted tissue at each time point after application of the graft to the subcutaneous layer of the rabbit's back. Based on the results, the clinical usefulness of Gore Bio-A as an alternative soft tissue was established.

## 2. Material and Methods

**Figure 1:**
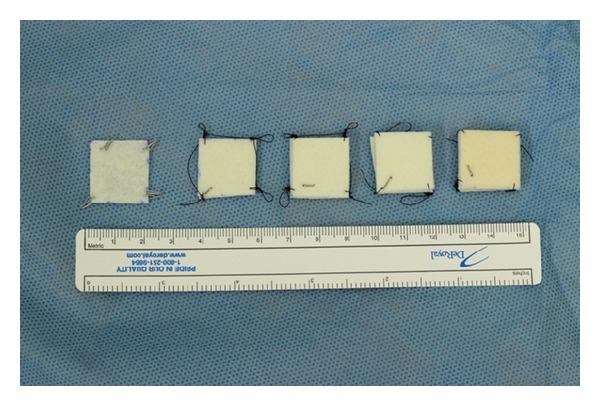
Gross appearance of Gore Bio-A product; 1 layer, 2 layers, 3 layers, 4 layers, and 5 layers (left to right).

This study was conducted with the authorization of the IACUC (Institutional Animal Care and Use Committees) from an affiliated institution of the author and with the observance of laboratory animal ethics regulations. The laboratory animals used were six male New Zealand rabbits that were 2.5 kg in weight. The laboratory animals were bred in the same conditions of fixed temperature, humidity, and assorted feed. The Gore Bio-A used was square in shape (1.5 mm thick and 20 mm wide and long), and it was prepared in one to five layers. To fix a layer, the corners of Gore Bio-A were sutured using nonabsorbable sutures and stapled to mark the absorption during the test (Figure 1). The premade implant was sterilized with EO (ethylene oxide) gas and sealed after drying. The rabbits were anesthetized with a 1 : 1 mix of Zoletil and Rompun that was injected into the muscle of the femoral region at a dose of 1 cc/kg, and additional anesthesia was performed to maintain anesthesia in case of shortage. In order to hold the rabbits on the holder, aseptic conditions were created by removing the hairs on the back using a clipper and disinfecting the area with 10% povidone-iodine solution and covering it with an OP drape.

**Figure 2:**
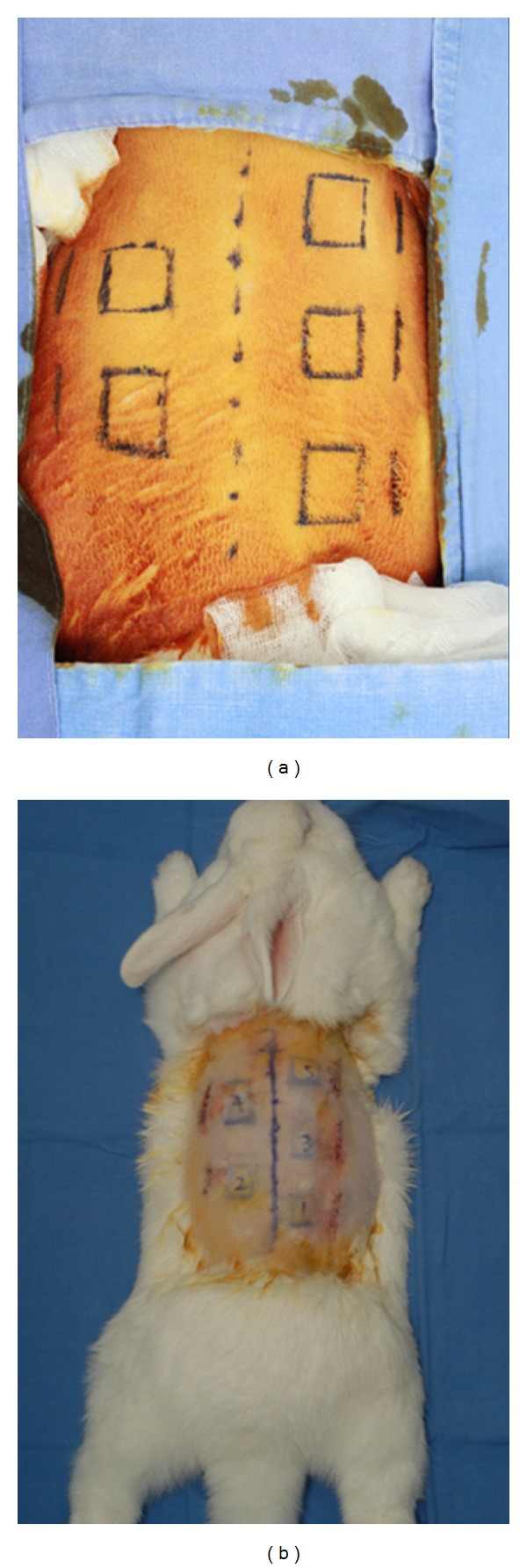
(a) Preoperative design on the back of the rabbit. (b) Immediate postoperative view.

Based on the midline drawn on the back of the rabbit, two or three sites were designed for Gore Bio-A insertion at both sides. Two cm incision lines were drawn 1.5 cm from the distal line of the squares. Lidocaine was injected as a local anesthetic, and the incision was made using a Blade 15 for sublation. The subcutaneous layer was exposed, and the prepared Gore Bio-A was inserted in the sites in one to five layers. To prevent the change in the initial location, the Gore Bio-A was sutured using Monosyn 4-0, and the incision wound was sutured using Nylon 4-0 (Figure 2).

The operation wound was washed with saline solution and hibitane solution and terramycin ointment after suture.

For infection prevention in each rabbit, 50 mg/kg of an antibiotic (cefotaxime) was injected into the muscle two times a day from the day of the operation to two days after the operation, and the infection status and inflammation symptoms were checked while performing wound disinfection once every five days. After transplantation, two rabbits at one, three, and six months were sacrificed with a KCL injection into the vein of the rabbits under anesthetic conditions with an anesthetic injection, and the graft was collected including the surrounding tissue. The collected implant was observed macroscopically for its relation and adhesion to the surrounding tissue and its shape. Then, each tissue was observed for histological changes including the inflammation response, ingrowth of the surrounding soft tissue, collagen synthesis level, neovascularization, and foreign body reaction status with respect to time using an optical microscopic and the standard method of paraffin embedding and H&E (hematoxylin and eosin) staining after fixation with 10% formalin. Each section was scored for each item, with 3 points given for over 65%, 2 points for 25–65%, 1 point for below 25%, and 0 points for no observance.

## 3. Results

### 3.1. Gross Findings (Figures 3, 4, and 5)

In all the groups with grafted Gore Bio-A, no complication, including seroma, infection, or inflammation, was found on gross observation. The grafted single layer of Gore Bio-A showed severe disfiguration, changing from its square shape, and Gore Bio-A became adherent enough to the surrounding tissue to make separation from the group difficult a month after its grafting ((a), (b)). However, a month after the grafting, the two- to five-layer grafted groups maintained their figure, and the grafted Gore Bio-A was easily separated from the surrounding tissues. In the groups, three months after the grafting ((c), (d)), the shapes of the one- and two-layer grafted groups were not maintained, and one layer was completely absorbed in the tissue, so the figure was difficult to identify. As for the three- to five-layer grafted groups, their square shapes were reasonably maintained but their adhesion to the surrounding tissues was severe and they were not able to be detached from the tissues. Six months after the grafting ((e), (f)), most of the grafted materials in the groups were absorbed. As for the one- and two-layer grafted groups, the grafted Gore Bio-A could hardly be distinguished from the surrounding tissue (it was identified based on the marked staples). The three- to five-layer grafted groups did not maintain their initial shapes and were almost absorbed completely into the surrounding tissues.

### 3.2. Histological Findings

Histological results of the grafted Gore Bio-A are presented in Tables 1 and 2. Microscopic findings demonstrated progressive neovascularization, fibroblast infiltration, and new collagen formation over time. Six months after implantation, Gore Bio-A was almost absorbed and degenerated, not maintaining its volume.

#### 3.2.1. One Month after the Gore Bio-A Grafting (Figure 6)

Mild inflammatory cells infiltration around the grafted Gore Bio-A was observed, and a slight foreign body reaction was detected.

The fusion between preexisting connective tissue and Bio-A (the ingrowth of Bio-A) was identified, and moderate neovascularization was observed. We can also find the deposition of stromal collagen. There was no significant difference in the histological reactions according to the degree of Bio-A insertion. The thickness of the grafted material was 4.5 mm (4.5, 4.5) for one layer because the tissue was thicker due to the ingrowth of the connective tissue than the inserted Bio-A. The results were 3.5 mm (3.5, 3.5) for two layers, 4.75 mm (4.5, 5.0) for three layers, 6.0 mm (6.0, 6.0) for four layers, and 8.25 mm (8.0, 8.5) for five layers.

#### 3.2.2. Three Months after the Gore Bio-A Grafting (Figure 7)

The number of inflammatory cells decreased around the Gore Bio-A and showed mild foreign body responses. There was no difference in terms of neovascularization, while the density of the collagen stroma and ingrowth of connective tissue were increased. The ingrowth of connective tissue and the new vessel formation showed a steady increase according to the thickness of the inserted graft. Measured thicknesses of the grafts were 4.25 mm (4.5, 4.0) for 1 layer, 4 mm (4.5, 3.5) for 2 layers, 4.25 mm (3.5, 5.0) for 3 layers, 5.25 mm (5.0, 5.5) for 4 layers, and 6.0 mm (5.5, 6.5) for 5 layers.

#### 3.2.3. Six Months after the Gore Bio-A Grafting (Figure 8)

Much of the grafted Gore Bio-A was absorbed and giant cells were sporadically observed between the newly formed soft tissues. From one to five layers, the grafted materials were mostly replaced with soft tissue, and the structure and volume of the inserted Bio-A were not maintained but were fully absorbed by the tissue.

## 4. Discussion

Using autologous tissue is the most ideal method for correcting dented or changed soft tissue in congenital malformation or injury or supplementing a shortage of tissue during aesthetic operations.

However, it is difficult to predict results according to the risk of resorption and scarring. And it has the potential of causing complications at the donor site, and the amount that can be harvested is limited [1–3]. Research on many new substances and many tests of usefulness has been made, and many products are in use. The conditions for an ideal implant of soft tissue substitution are that the implant is easy to obtain, has good biocompatibility, provokes no immune response after transplantation, has enough durability to maintain the volume for a long time, and enables the prediction of the soft tissue volume after insertion [4, 5].

The currently developed allograft is acellular dermis matrix such as Alloderm, which is widely used for wound recovery, with a sublayer skin graft used for burns patients. The range of use of Alloderm recently has been increasing, with its use as a soft tissue substitute for transplantation under the skin [6].

When Alloderm is transplanted subcutaneously, it can substitute for collagen, with neovascularization on all layers achieved in a comparatively short period of time even if there are differences in speed according to the thickness after transplantation. However, it has the limitation of use that the volume is not maintained for a long time and the costs of manufacturing it are high.

The artificial implant can be divided into absorbable and nonabsorbable substances, and a nonabsorbable substance, the silicone implant, is widely used. Silicone began to be used from the 1960s and since then it has become a widely used prosthesis in medical practice, with a low price, ease of handling, and ease of removal in the case of side effects such as infection. However, immoderate velum formation, spread and protrusion of the implant, shortage of internal growth as an implant of tissue, and biorejection may occur [4, 7].

There have been many studies on porous materials for the development of ideal implant materials by supplementing the shortcomings of artificial materials. Gore-Tex was one of the implant materials that were developed as porous materials in the late 1960s, and its safety has been proven with its use in vascular surgeries for 20 years. It was also approved by the FDA in 1993 for use in cosmetic surgery, including rhinoplasty [8]. Morphologically, it has a woven nodule-fibril structure by the connections between the thin and elastic flexible fibrils of polytetrafluoroethylene (PTFE) with the solid nodules of PTFE. The hollow space is taken up by air, which allows the surrounding connective tissue to grow inward. As a result, the implanted materials are easily fixed and stabilized, and film formation and shrinkage can be minimized [5]. However, PTFE is a nonabsorbable material, and there are various clinical reports of its side effects such as infection, malpositioning, protrusion, seroma, and persistent swelling [7, 8].

Gore Bio-A, an absorbable porous implant material, was recently developed and used for hernia or anal fistula [9–13]. As it has good biocompatibility due to its porosity and as it is absorbable, the authors expected fewer disadvantages of using this artificial material. On this basis, this study on Gore Bio-A as an alternative to artificial materials as an implant material for soft tissue augmentation was designed. Gore Bio-A has a porous, three-dimensional (3D) woven polymer structure that consists of polyglycolic acid (PGA) and trimethylene carbonate (TMC). Previous studies reported that this structure works as a scaffold and stimulates the regeneration and healing of tissue via deposition of cells and neovascularization of soft tissue. The studies further reported that the material itself was absorbed slowly for six months and new tissues replaced the space [9, 10]. The authors expected that the maintained volume of the newly grown soft tissue with the replacement of the grafted Gore Bio-A could augment the soft tissue outcome. As such, the replacement of the soft tissue, the degree of absorption, and the volume change according to the grafted period and the multiple graft layers were investigated. The results of this study showed that more Gore Bio-A was absorbed in the surrounding soft tissues when the grafting period was longer and the layer was thinner, based on naked-eye observation. The surrounding tissue and the grafted material were separate up to one month after the grafting but were completely fused after three months. Six months after the implant, the absorption was completed and the border of the grafted material and the surrounding soft tissue could hardly be distinguished, which showed that Gore Bio-A had good biocompatibility with tissues. The previous studies reported that a reasonable volume of the material was maintained after its absorption and was replaced with newly grown tissue, but the volume and shape of the grafted material were severely changed after its absorption in this study. There are some experiment reports that infiltration of inflammatory cells was observed on day 3, collagen and vessel formation in the wound healing process on day 7, and the start of the maturation of the formed collagen and the absorption of the grafted materials on week 2. Ingrowth of the surrounding soft tissue was observed in the Gore Bio-A based on the initial histological findings of the material in the abdomen of a rabbit. The histological findings from this study showed ingrowth of the surrounding soft tissue, collagen fiber formation, inflammatory cells in the surrounding areas, and a foreign body reaction one month after the grafting. The results did not show a significant difference according to the thickness of the implanted materials. Three months after the grafting, the space left after Gore Bio-A was absorbed was replaced with the surrounding soft tissues and mature collagen fibers. Six months after the grafting, Gore Bio-A was completely absorbed and replaced with the tissues, regardless of the grafted layers. However, the grafted volume was not maintained, unlike in the previous studies, as mentioned earlier.

It was found that Gore Bio-A has ideal qualities as an alternative implant material such as biocompatibility and good replacement with its surrounding tissues but that it also has a disadvantage in that it does not maintain the grafted volume. As Gore Bio-A has biocompatibility and can be absorbed while hypothetically maintaining its grafted thickness, further methodological studies to investigate how it maintains its thickness and qualitative studies on the grafted thickness should be conducted. Also, continuous attempts to find other alternative materials that can keep the volume of the grafted materials and have biocompatibility should be made.

## 5. Conclusion

This study aimed to investigate the changes in the eminence of Gore Bio-A and histological changes according to the time after the multilayer grafting in the back of a rabbit. The conclusion of this study is as follows. This absorbable Gore Bio-A is considered beneficial in terms of its stability and replacement by the surrounding soft tissue. However, it absorbs and replaces the tissue without maintaining the grafted volume, so it is considered inapplicable for use for soft tissue augmentation, as it was first intended to be used.

## Figures and Tables

**Figure 3:**
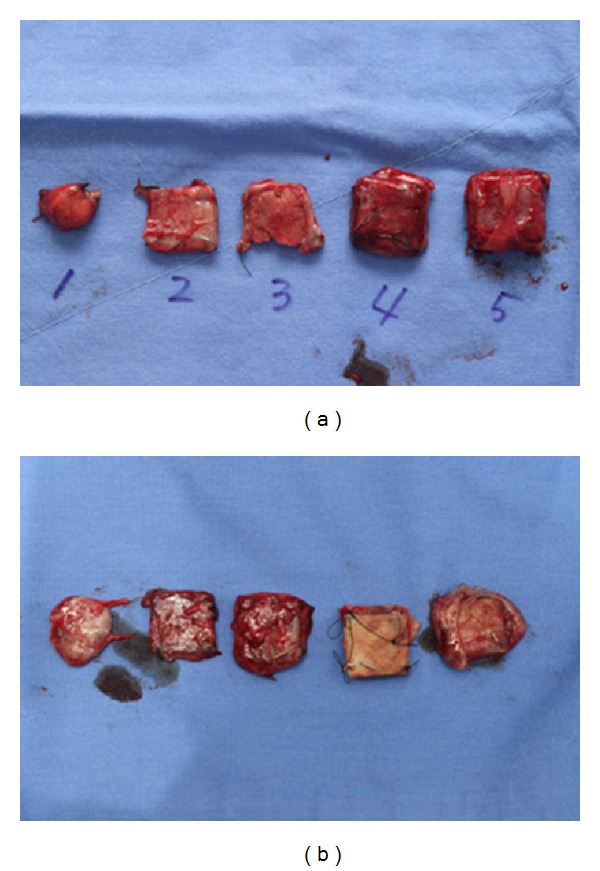
Gross appearance of inserted Gore Bio-A after 1 month ((a)/(b)).

**Figure 4:**
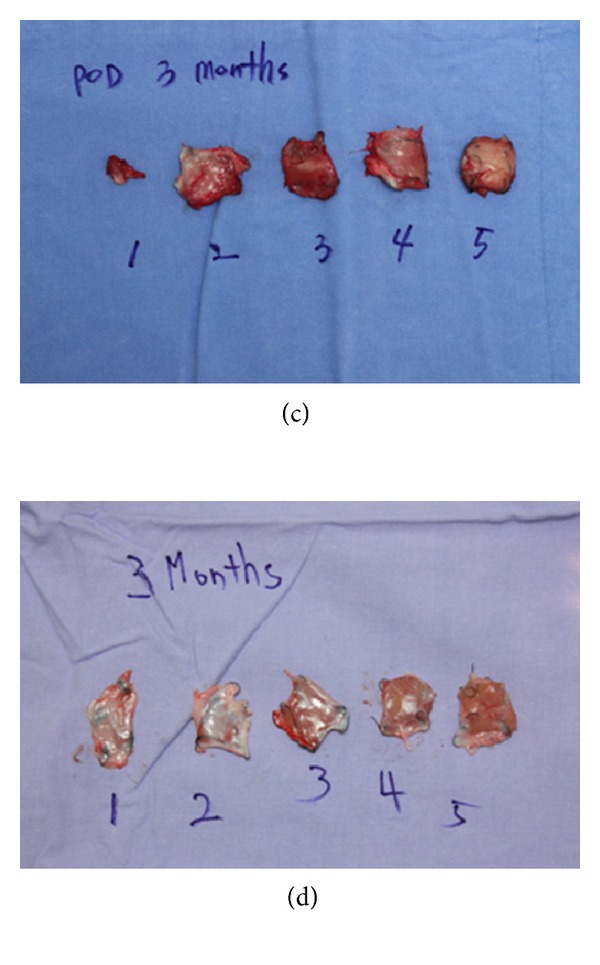
Gross appearance of inserted Gore Bio-A after 3 months ((c)/(d)).

**Figure 5:**
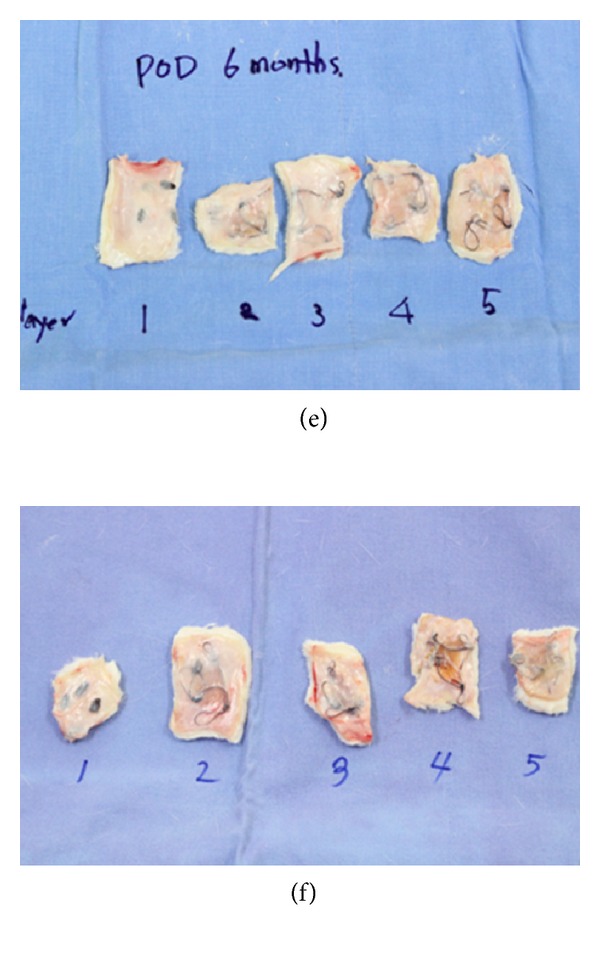
Gross appearance of inserted Gore Bio-A after 6 months ((e)/(f)).

**Figure 6:**
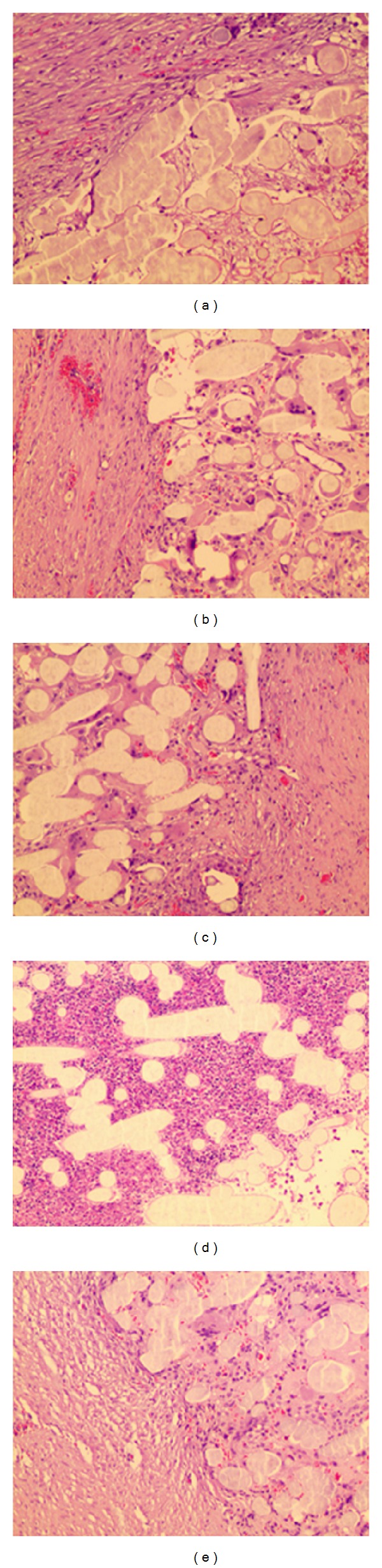
Microscopic findings of implanted Gore Bio-A 1 month after operation (H&E stain, ×100) ((a) to (e)) 1 layer to 5 layers.

**Figure 7:**
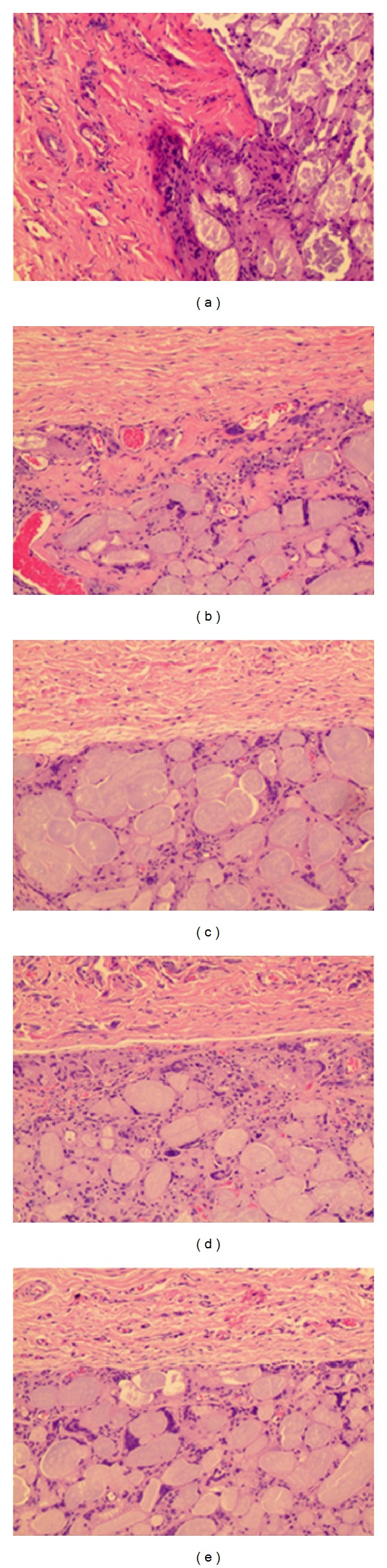
Microscopic finding of implanted Gore Bio-A 3 months after operation (H&E stain, ×100) ((a) to (e)) 1 layer to 5 layers.

**Figure 8:**
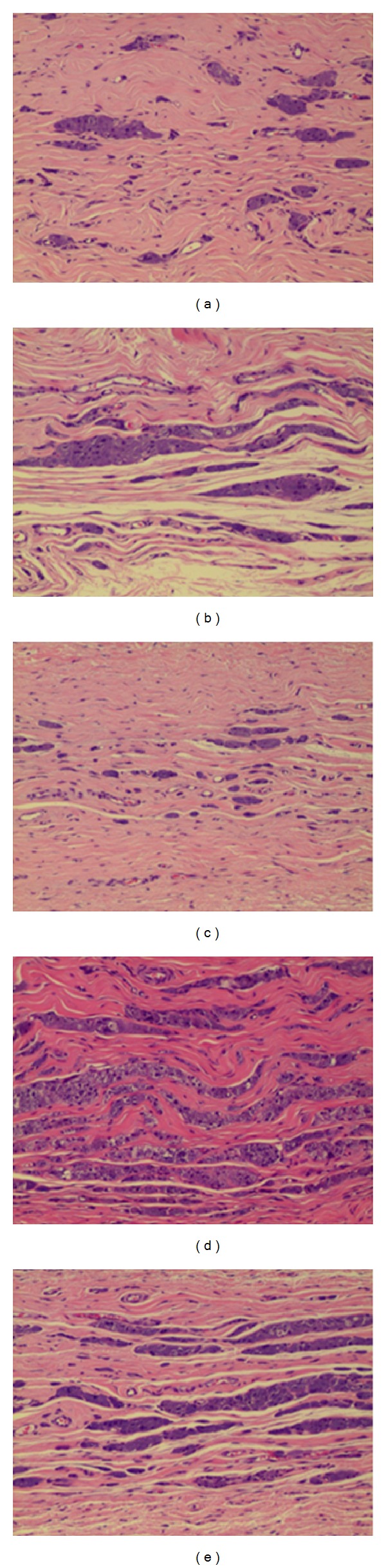
Microscopic findings of implanted Gore Bio-A 6 months after operation (H&E stain, ×100) ((a) to (e)) 1 layer to 5 layers.

**Table 1:** The change of mean thickness of Gore Bio-A with time (mm).

Bio-A	Period				Ratio*		
	Preoperative thickness	1 month	3 months	6 months	1 month	3 months	6 months
1-layer	1.5	4.5	4.25	0.54	300%	283%	36%
2-layer	3.0	3.5	4.0	0.64	117%	133%	21%
3-layer	4.5	4.75	4.25	0.60	105%	94%	13%
4-layer	6.0	6.0	5.25	0.48	100%	86%	8%
5-layer	7.5	8.25	6.0	1.06	110%	80%	14%

*Mean thickness (%) at 1, 3, and 6 months compared to the preoperative thickness.

**Table 2:** Microscopic findings according to the duration of Gore Bio-A implantation.

	Inflammatory cell infiltration*	Connective tissue ingrowth	Collagen deposition	Foreign body reaction	Neovascularization
1 month ((a)/(b))					
1-layer	0/1	3/3	2/3	3/3	2/1
2-layer	0/0	2/2	2/3	3/3	2/2
3-layer	0/1	3/3	3/3	3/3	3/2
4-layer	2/1	1/3	1/3	1/3	1/2
5-layer	1/1	3/3	3/3	3/3	3/2
3 months ((c)/(d))					
1-layer	0/0	3/2	3/1	3/1	3/1
2-layer	0/0	2/2	1/1	3/2	2/2
3-layer	0/0	2/2	1/1	3/2	2/2
4-layer	0/1	2/2	1/1	3/2	2/2
5-layer	0/1	2/2	3/2	3/2	1/2
6 months ((e)/(f))					
1-layer	1/1	3/3	1/1	2/1	1/1
2-layer	1/1	3/3	0/0	3/2	1/1
3-layer	1/1	3/3	0/0	2/1	1/1
4-layer	1/1	3/3	0/0	3/2	1/1
5-layer	1/1	3/3	1/0	2/3	1/1

Scoring: 0: negative; 1: mild; 2: moderate; 3: severe.

*0: no inflammatory cell infiltration; 1: focal inflammatory cell infiltration; 2: acute suppurative inflammation.
